# Sexually Selected Male Plumage Color Is Testosterone Dependent in a Tropical Passerine Bird, the Red-Backed Fairy-Wren (*Malurus melanocephalus*)

**DOI:** 10.1371/journal.pone.0026067

**Published:** 2011-10-05

**Authors:** Willow R. Lindsay, Michael S. Webster, Hubert Schwabl

**Affiliations:** 1 School of Biological Sciences and Center for Reproductive Biology, Washington State University, Pullman, Washington, United States of America; 2 Cornell Lab of Ornithology and Department of Neurobiology and Behavior, Cornell University, Ithaca, New York, United States of America; University of Lethbridge, Canada

## Abstract

**Background:**

Sexual signals, such as bright plumage coloration in passerine birds, reflect individual quality, and testosterone (T) may play a critical role in maintaining signal honesty. Manipulations of T during molt have yielded mixed effects on passerine plumage color, in most cases delaying molt or leading to production of drab plumage. However, the majority of these studies have been conducted on species that undergo a post-nuptial molt when T is low; the role of T in species that acquire breeding plumage during a pre-nuptial molt remains largely unexplored.

**Methodology/Principal Findings:**

We experimentally tested the effects of increased T on plumage color in second-year male red-backed fairy-wrens (*Malurus melanocephalus*), a species in which after-second-year males undergo a pre-nuptial molt into red/black (carotenoid and melanin-based) plumage and second-year males either assume red/black or brown breeding plumage. T treatment stimulated a rapid and early onset pre-nuptial molt and resulted in red/black plumage acquisition, bill darkening, and growth of the sperm storage organ, but had no effect on body condition or corticosterone concentrations. Control males molted later and assumed brown plumage. T treated males produced feathers with similar but not identical reflectance parameters to those of unmanipulated after-second-year red/black males; while reflectance spectra of red back and black crown feathers were similar, black breast feathers differed in UV chroma, hue and brightness, indicating a potentially age and plumage patch-dependent response to T for melanin- vs. carotenoid-pigmentation.

**Conclusions/Significance:**

We show that testosterone is the primary mechanism functioning during the pre-nuptial molt to regulate intrasexually variable plumage color and breeding phenotype in male red-backed fairy-wrens. Our results suggest that the effects of T on plumage coloration may vary with timing of molt (pre- vs. post-nuptial), and that the role of T in mediating plumage signal production may differ across age classes, plumage patches, and between pigment-types.

## Introduction

Condition-dependent regulation of sexually selected display traits is a critical assumption of honest advertisement models of sexual selection, as these traits impact the frequency and outcome of social interactions and can ultimately determine reproductive fitness [1], [2] Testosterone (T) regulates many male reproductive traits and is a potential mechanism for enforcing signal honesty [3] as heightened T can carry concomitant costs (immunity [3] (but see [4]), metabolism [5], behavior [6]) that may differentially effect fitness depending on male quality [7]–[9]. However, the role of T in maintaining signal honesty is controversial [4], in part because not all sexual signals, particularly plumage color in birds, appear to be regulated by T [10].

Avian plumage color is a highly variable, multifunctional signal of male quality [11]–[13] and thus should be under rigorous social and physiological control to deter cheating. However, although T is known to stimulate sexual dichromatism (i.e., acquisition of bright, male-typical plumage) in the avian order Charadriiformes, T is not thought to function in this capacity in other avian orders [14].There is, however, increasing speculation that T may mediate intrasexual variation in plumage color, even for species where sexual dichromatism is not T-dependent (reviewed by [14]). In particular, some recent studies have focused on the role of T in regulating color patterns of male birds in the order Passeriformes, where the selective advantages and condition dependence of plumage elaboration has received extensive research interest. In these studies, the effect of T on control of intrasexually variable passerine plumage color has found mixed support (supporting: [15]–[17], opposing: [18]–[21]).

Discrepancies between species in the effects of T on induction of nuptial plumage color might be explained by differences in the timing of molt, in particular, its temporal relationship to onset and end of reproduction. That is, the likelihood that T functions during molt to directly affect resulting feather coloration is expected to be highest in those species that obtain their nuptial plumage during a pre-nuptial molt that occurs immediately prior to breeding when gonads actively produce T (e.g., Superb fairy-wren, [22]; but see [23]). Notably, most passerines for which the role of T in plumage color has been studied are seasonal breeders in the temperate/high latitude zones that acquire nuptial plumage during a single annual post-nuptial molt, which occurs after gonads have regressed and T levels are low [16], [17], [19], [20], [24]. In these species, experimentally elevated T delays molt [19], [20], [25]–[28] and/or leads to the production of drab plumage [20]. In some of these species, though, T regulates intrasexual color variation outside of the molting period by mediating behaviors, such as preening, that influence plumage appearance [16], [17], or by altering developmental processes via the effects of yolk androgens with resulting impacts on adult plumage color [29], [30]. Little is known about direct effects of T on feather coloration during molt for passerine species that undergo a pre-nuptial molt.

We experimentally tested the role of T in production of variable male nuptial plumage in a pre-nuptial molting tropical passerine, the red-backed fairy-wren (*Malurus melanocephalus*). Variation in male red-backed fairy-wren nuptial plumage is sexually selected with second-year males (SY; age 1 yr) acquiring either an elaborate red/black nuptial plumage typical of older males, or female-like brown plumage; plumage color affects their reproductive success [31], [32]. The red/black and brown phenotypes differ in body morphology with red/black males having darkened bills and enlarged cloacal protuberance (CP) sperm storage organs as compared to brown males; both bill color and CP volume are positively associated with heightened androgen concentrations [33], [34]. Male red-backed fairy-wrens experience large seasonal fluctuations in T (contrasting with many tropical species, see [35], [36]), with androgen concentrations during the pre-nuptial molt as high as during breeding [33]. Molting male androgen concentrations are positively correlated with condition as well as resulting feather coloration, suggesting an honesty enforcing role for T in the regulation of nuptial plumage color [33]. Indeed, a critical role for T in regulation of plumage coloration has already been established for the congeneric Superb Fairy-wren, to our knowledge the only passerine species for which experimental evidence clearly demonstrates T-dependent sexual dichromatism ([22], but see [37] indicating additional genetic components). Red-backed fairy-wrens also provide a unique opportunity to simultaneously test for within-individual effects of T on different pigment types comprising the elaborate nuptial plumage: red carotenoid-pigmented back feathers and black melanin-pigmented body feathers [38]. While T is increasingly thought to regulate melanin-based plumage color (reviewed by [39], [40]), and experimental evidence suggests that T can stimulate carotenoid transport [41]–[43] and influence carotenoid-based coloration of fleshy integument [42], [44]–[46], to our knowledge a connection between T and carotenoid-pigmented plumage coloration is yet to be shown [14].

Using testosterone (T) and control implants, we assessed the effects of T on 1) onset of the pre-nuptial molt, 2) red/black plumage production, 3) the reflectance parameters of carotenoid- and melanin-pigmented nuptial plumage produced by unmanipulated and T treated males, 4) potential costs of elevated T (body condition and concentrations of the stress hormone corticosterone), and 5) changes in additional secondary sex characters (bill color and CP volume). Our results unequivocally demonstrate a pivotal role for T in regulation of male red-backed fairy-wren breeding phenotype, including plumage color.

## Methods

### Ethics Statement

All captured and implanted birds were treated in a safe and human manner. Silastic implants were set sub-cutaneously under the skin of the back, a location shown to reduce interference with connective tissue and chances of skin rupture [47]. As Red-backed Fairy-wrens show high sight fidelity with defined territories, we were able to recapture all but four experimental birds (93% recaptured) to remove empty implants; birds that were not recaptured most likely dispersed from the study site. Previous research on other passerines has shown that long term maintenance of empty implants (implants from which all hormone has diffused) have no effect on survival [48]. All procedures were approved by the Institutional Animal Care and Use Committee (protocol no. 3067) of Washington State University, the James Cook University Animal Ethics Review Committee (approval no. A1004), and the Queensland Government Environmental Protection Agency.

### Study Species and General Methods

We studied a color-banded population of the common cooperatively breeding red-backed fairy-wren near Herberton, Queensland, Australia (145°25′E, 17°23′S) during the austral spring of 2007 (August–October). We target trapped adult birds using mist nets, weighed each bird to the nearest 0.1 grams, estimated age from previous history or from skull ossification (ossification scale modified from [49]), and took a series of standard morphological measurements including: proportion of the body covered in nuptial plumage, molt score, measurement of the CP, bill color, and tarsus length. We scored molt visually on six body regions (head, back (including nape and mantle), wing, tail (rectrices only), belly, and chest (including chin, throat, and breast)) as none (0), light (1), medium (2), or heavy (3) based on the proportion of feathers in pin (actively growing feathers encased in a feather shaft, ie. “pin feathers”). A bird was considered to be molting when the cumulative molt score was 2 or more. In no case were the pins resulting from feather plucking at previous captures (see “experimental methods”) considered in the molt score. Birds were placed in holding bags with plastic inserts for collection of fecal samples. From each captured bird we collected a maximum of 80 µl whole blood; we separated plasma from red blood cells and stored both plasma and faeces in liquid nitrogen until transport to Washington State University where it was kept at −20°C awaiting further analysis.

Red-backed fairy-wrens are seasonally sexually dichromatic with both males and females assuming a similar brown plumage for the duration of the non-breeding season (a few after-second-year males (ASY; age 2+ yrs) express nuptial plumage year round; pers. obs.). All birds undergo a pre-nuptial molt that occurs directly prior to the onset of breeding (August–October; Lindsay et al. 2009). During the pre-nuptial molt, all ASY males assume a red/black nuptial plumage consisting of red carotenoid-pigmented feathers [38] on the back and scapulars, and black (presumably melanin-based) feathers on the head, tail, belly, chest, and outer wing coverts (see photographs in [50]). Primary and secondary wing feathers are brown on all birds. SY males can either assume the red/black nuptial plumage (15% of SY males; [50]) or a female-like brown plumage with brown on all body regions excepting a white breast and upper belly. Although the distribution of plumage color (red/black vs. brown) across the population of breeding males is bimodal [32], a small proportion of males assume an intermediate coloration. Thus, we calculated the amount of the body covered in red/black nuptial plumage by summing plumage coloration scores from five body regions (head, back, tail, belly, chest), each assessed using a scale from 0–10 where 0 indicated the absence of any red or black feathers and 10 indicated a plumage patch consisting of all red/black feathers. This summation gave a maximum score of 50 (for an entirely red/black bird), and so we converted this score to a percentage (% red/black; equivalent to “% brightness” in [32], [33]) by doubling the summation.

Acquisition of male nuptial plumage during the pre-nuptial molt coincides with a darkening of the bill [51] and growth of the CP, a prominent sperm storage organ in the Maluridae [31]. To measure CP size, we took three measurements of the posterior protuberance: length (L), width (W) and depth (D). From this we calculated CP volume as volume of a cylinder using the formula π×D/2×W/2×L [31], [52]. Bill color is an ostensibly androgen sensitive signal of breeding status in this species [33], [34], [51], ranging from pale cream to ebony black. We visually scored bill color in each of four sections of the bill (top and bottom, anterior and posterior; cumulative bill color score ranges from 1–40) on a scale of 1–10 and calculated overall bill color as the sum of these scores. To minimize subjectivity, we designed a “bill color ruler” from standardize digital photos (against a color checker embedded in each photo) of a range of red-backed fairy-wren bills covering the spectrum from cream to black.

### Experimental Methods

We randomly assigned SY birds to either a testosterone (crystalline testosterone – Sigma T1500) implant group (“T males”; N = 8) or a control group of birds that received an empty implant (“control males”; N = 7). Due to an inability to differentiate SY males from females, we implanted both sexes and determined sex post-hoc using standard molecular genetics techniques ([53]; final sample sizes listed above are for implanted males). We also assigned birds to an anti-testosterone treatment group (ATD/flutamide, see methods Text S1; “Anti-T males”; N = 7), but due to implant loss (all Anti-T males lost implants within two weeks of implantation), this treatment was excluded from primary analyses and the effects of Anti-T treatment are discussed in the supplementary appendix (Text S1,Table S1, Fig. S1, Fig. S3, Fig. S5).

We scaled our silastic (Dow Corning) implants to an effective length of 4–5 mm, inner diameter of 1.47 mm and outer diameter of 1.96 mm (as in [22]) and sealed their ends with Silicone Adhesive (MED-1037, NuSil Silicone Technology). We set implants sub-cutaneously on the back adjacent to the back feather tract, inserting each implant into a small (1–2 mm) skin incision which was sealed with veterinary skin adhesive and a small piece of OpSite flexible wound dressing. We set implants at the first sign of molt in the study population (the first pin feather seen on a captured adult red-backed fairy-wren) and removed implants after the completion of molt (∼1 month from implantation; see below).

We captured each implanted bird up to three times post-implantation (implantation occurred between Aug. 21-Sept. 6) in order to assess changes in molt, plumage color, hormone levels, and body morphology. We conducted a “mid-treatment” recapture (Sept. 2–Sept. 21) between 15 and 24 days post-implant (mean = 17.8 days; T N = 6, control N = 6), removed implants (Oct 3–Oct. 19) between 35 and 56 days post-treatment (mean = 45.2 days; T N = 6, control N = 6), and conducted a “post-treatment” recapture (Dec 26–Jan 4) between 115 and 130 days post-implantation (mean = 122.1 days; T N = 3, control N = 3). We simultaneously assessed changes in unmanipulated ASY male coloration and morphology in order to determine whether effects of T treatment paralleled seasonal changes documented in older males naturally expressing enhanced plumage coloration (100% red/black) and body morphology. ASY measurements were divided post-hoc into four time categories corresponding to the four experimental trapping periods (Aug 21–Sept 6, ASY N = 12; Sept 7–Sept 21, ASY N = 16; Sept 22–Oct 19, ASY N = 18; Dec 26–Jan 4, ASY N = 2).

At implantation we plucked 6–10 feathers from each of three body regions (back, crown, and breast) to standardize feather growth under the influence of varying treatments, and re-plucked feathers from these same body regions when implants were removed for subsequent analysis of reflectance parameters (see below). In addition to our experimentally treated SY males, we also plucked feathers from 4 unmanipulated ASY males captured during a similar time period.

### Reflectance Spectrometry and Color Analysis

We used spectrographic analysis to objectively quantify differences in plumage color between treatment types (T, control) and un-manipulated ASY males. We stored feathers collected at implant removal in glassine envelopes and, after transport to Washington State University, mounted 6–10 overlapping feathers from the same body region (back, breast or crown) on black construction paper. We used an Ocean Optics USB2000 UV-VIS spectrometer (R200-7UVVIS probe and PX2 pulsed xenon light source; for more details see [54]) to obtain four measurements of reflectance (R) in each plumage patch for each individual. Using the program CLRv1.05 [55], we calculated tri-stimulus color variables (spectral intensity, spectral purity, spectral location) across all wavelengths (λ) of the avian visual spectrum (320–700 nm); we collapsed our repeat measurements into a single average spectrum per individual and plumage patch. We summarized our reflectance spectra with 5 color metrics: 1) total reflectance or brightness (R_total_ = sum of R between 320–700 nm), 2) hue (λ_Rmax_), 3) spectral purity or chroma ((R_max_-R_min_)/R_mean_), 4) saturation in the ultraviolet range of the spectrum or UV chroma (R_320–400 nm_/R_total_), and 5) red chroma (i.e., saturation in the red range of the spectrum, R_625–700 nm_/R_total_).

### Plasma and Fecal Steroid Radioimmunoassay

We utilized plasma and fecal samples of treated SY and un-manipulated ASY males to assess changes in circulating and excreted concentrations of androgens and corticosterone. Our radioimmunoassay for total plasma androgen concentration followed previously published protocols [33], [56] with some modification in order to conduct simultaneous direct measurements of corticosterone concentrations (without column chromatography). Steroids were extracted from plasma with two consecutive 4 ml washes of diethyl-ether and were not further purified. We added 200 µl phosphate-buffered saline with gelatine, pH 7.1 (PBSg) to dried extracts. 100 µl of re-dissolved extract was placed directly into singlet androgen assay vials and 20 µl into individual corticosterone recoveries (containing recovered fraction of initial 2000 cpm tritium labelled corticosterone; mean corticosterone recovery was 79.17%). We added an additional 200 µl PBSg to the remaining 80 µl of re-dissolved steroid extract and prepared duplicate 100 µl corticosterone assay tubes for all samples. Four androgen recovery samples containing 2000 cpm tritium-labelled testosterone were run per assay with no simultaneous measurement of corticosterone (mean recovery of 91.28%). Radioimmunoassays were conducted using tritium-labelled testosterone (PerkinElmer Life Sciences NET-553, Waltham, Massachusetts USA) and corticosterone (PerkinElmer Life Sciences NET 399) with testosterone and corticosterone antibodies (testosterone antibody; Wien Laboratories T-3003, Flanders, New Jersey USA; corticosterone antibody; Esoterix Endocrinology B3-163) and according to standard techniques [56]. Total androgen concentrations are reported as pg/ml and corticosterone as ng/ml. The average intra-assay coefficient of variation across the three assays was 3.47% for androgens and 13.69% for corticosterone and the inter-assay variation was 14.95% for androgens and 14.93% for corticosterone (calculated according to [57]; androgen intra-assay variation calculated using 5 duplicate known samples per assay). The minimum detectable testosterone concentration given our assay parameters was 2.93 pg/ml and we detected testosterone between the ranges of 92.7 pg/ml and 5,127.1 pg/ml, mean = 722.2 pg/ml, median = 400.3 ml. The minimum detectable corticosterone was 1.17 ng/ml and we detected concentrations of 2.33 ng/ml to 110.5 ng/ml, mean = 41.3 ng/ml, median = 37.9 ng/ml. Samples from treatment types and sampling periods were distributed randomly across assays.

We conducted radioimmunoassay for total fecal androgen metabolite concentrations (modified from [58], [59]; as described and validated in [34]). In brief, we extracted steroid metabolites from lyophilized, pulverized fecal pellets using 1 ml of 75% ethanol in double-distilled water. After drying 500 µl of ethanolic extract at 40C° in a vacuum centrifuge, we added 200 µl of sodium acetate buffer containing β -glucuronidase/arylsulfatase to hydrolyse β-glucuronides and sulfate esters, which we then incubated for 16–18 hrs at 39 C. We ran duplicate assay tubes for each sample containing 20 µl hydrolyzed extract and 80 µl of PBSg. Assays were performed according to standard techniques [56] with mean recoveries of 74.3% and intra- and inter-assay coefficients of variation similar to those of previous studies [33], [34]. We detected fecal androgen concentrations of 33.5 pg/mg feces to 1,993.4 pg/mg with mean = 296.9 pg/mg and median = 108.8 pg/mg.

### Statistics

We used the Sheirer-Ray-Hare test, a nonparametric version of a two-way ANOVA modified from the Kruskal-Wallace test [60], to assess the influence of treatment (T, control, and un-manipulated ASY), time of capture (implantation, mid-treatment, and implant removal) and the interaction between treatment and time. We chose this analyses for two reasons: 1) our response variables (molt score, % red/black, CP volume, bill color, and androgen metabolite concentration) did not meet assumptions of normality, even after mathematical transformations; and 2) missing data due to incomplete sampling (individual's were trapped one or more but rarely all four times across the course of the season) lead us to reject repeated measures designs and accept the error associated with violating the assumption of independent sampling between time periods in an ANOVA. All pair-wise comparisons between treatment types at single trapping periods (implantation, mid-treatment, implant removal, or post-treatment) were either conducted using Kruskal-Wallace tests, or standard linear least-squares models. We used the residuals of a regression of mass on tarsus as our measure of body condition, a measurement which we have previously shown to correlate with fat stores and to provide similar results in analyses as use of mass or haematocrit [33]. All analyses were conducted using JMP 7.

## Results

### Androgens, Corticosterone, and Body Condition

Plasma androgen concentrations of T-treated males at implant removal (mean = 2,499±961 (SE) pg/ml, range 123–5,127 pg/ml, median = 2,417 pg/ml) were not significantly higher than those of un-manipulated pre-breeding males (ASY) sampled in the population (population mean = 798±174 pg/ml, range 157–1,550 pg/ml, median = 867 pg/ml, N = 9; χ^2^ = 0.89, df = 1, p = 0.346), and were physiological, as they fell well within the range of values documented for red/black males in our study population (pop mean across 7 years of sampling [2003–2009] = 981±60 pg/ml; range = 64–9,302 pg/ml, median = 512 pg/ml, N = 473). Concentrations of circulating androgens and excreted androgen metabolites were positively correlated (F_1,28_ = 53.84, p<0.0001, R^2^ = 0.657) and we obtained similar results from all major analyses using both, but due to incomplete sampling of plasma T (plasma from the mid-treatment recapture was utilized elsewhere), all analyses reported below were conducted with excreted fecal androgen metabolite concentrations.

**Figure 1:**
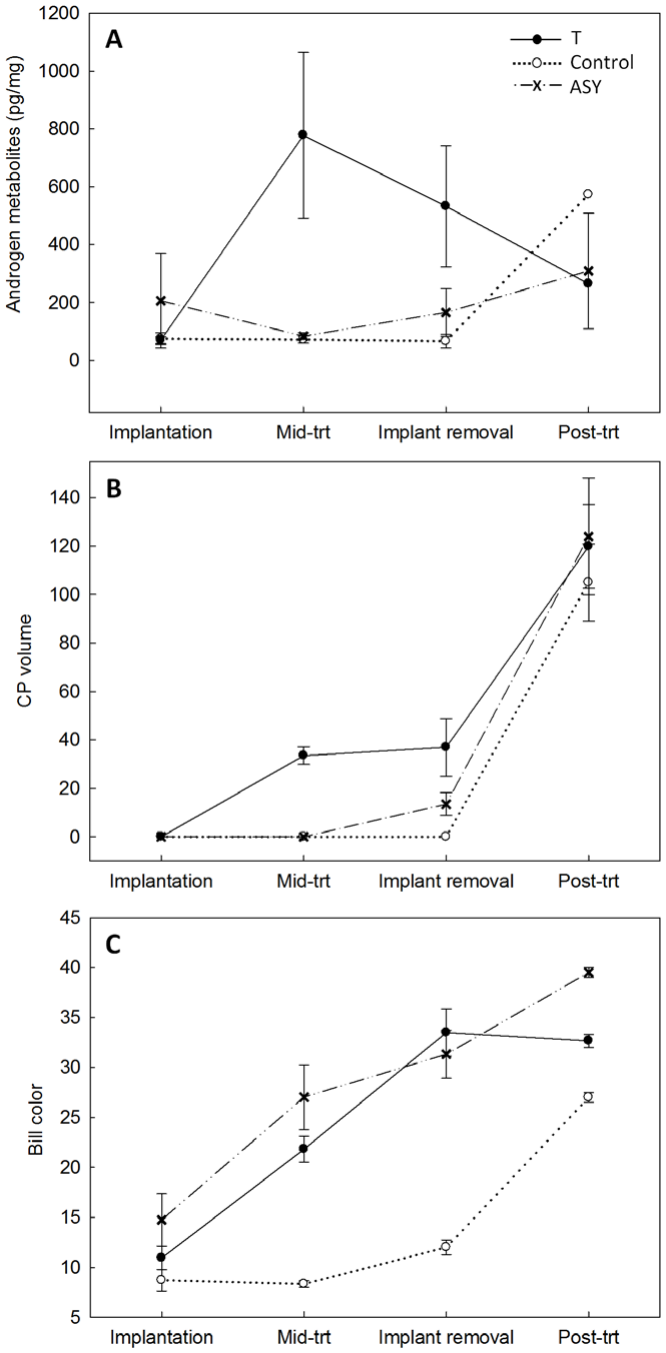
Treatment effects on androgen metabolite concentrations (A), cloacal protuberance volume (B), and bill color (C). Changes in mean response ± SE for second-year testosterone (T) and control implanted males, and unmanipulated after-second year (ASY) males at implantation, the mid-treatment recapture, implant removal, and the post-treatment recapture.

Excreted androgen metabolite concentrations varied with treatment (Table 1, Fig. 1A; see also Fig S1A) and changed across the course of the experiment in T males (χ^2^ = 11.01, df = 3, p = 0.012) with a dramatic increase following T-implantation and a decline after the implant was removed (Fig. 1A). Control, ASY, and T male excreted androgen metabolite concentrations were similar at the post-treatment recapture (Table 1, Fig. 1A).

**Table 1:** Effects of treatment (T, control, ASY), time since implantation (capture periods; implantation, mid-treatment recapture, and implant-removal), the interaction between treatment and time, and the long-term, within season consequences of treatment measured at the post-treatment recapture on male plumage characteristics, morphology, and excreted androgens.

	Treatment		Time		Trt×time		Post-treatment	
	H*, df	p	H, df	p	H, df	p	χ^2^, df	p
Molt score	6.23, 2	**0.044**	26.7, 2	**<0.0001**	6.95, 4	0.138	0.83, 2	0.659
% Red/black	11.6, 2	**0.003**	14.3, 2	**0.0008**	8.18, 4	0.085	7.06, 2	**0.029**
CP Volume	43.0, 2	**<0.0001**	12.7, 2	**0.002**	23.6, 4	**<0.0001**	0.00, 2	1.000
Bill color	22.4, 2	**<0.0001**	20.2, 2	**<0.0001**	6.17, 4	0.187	7.12, 2	**0.029**
Fecal T	9.55, 2	**0.008**	4.73, 2	0.094	5.12, 4	0.275	1.80, 2	0.407

*H∼χ^2^.

Statistically significant effects are indicated in bold.

T manipulation did not influence corticosterone levels, and also did not significantly affect body condition. T, control, and ASY males did not differ in plasma corticosterone concentration at either implantation (χ^2^ = 0.02, df = 2, p = 0.99; T (mean±SE) 39.58±6.31 ng/ml; control 35.09±5.33 ng/ml; ASY 33.54±7.21 ng/ml) or implant removal (χ^2^ = 0.42, df = 2, p = 0.81; T 29.69±7.79 ng/ml; control 24.55±7.5 ng/ml; ASY 49.41±13.73 ng/ml). Although there was a marginally non-significant difference in body condition between treatments (Fig S2; F_2,74_ = 3.06, p = 0.053), T male body condition was not significantly lower than that of ASY males, who were in significantly better condition than control males (Student's t; α = 0.05). Body condition did not change over the course of the experiment (Fig S2; F_2,74_ = 1.46, p = 0.239), and there was no interaction between treatment and time (F_4,74_ = 0.54, p = 0.709). At the post-treatment recapture there was no difference in body condition between treatments (F_2,6_ = 1.73, p = 0.255; Fig S2).

### Non-plumage Traits

T treatment induced premature cloacal protuberance (CP) development (Table 1, Fig. 1B); T males had measureable CP's by the mid-treatment recapture (mean volume = 33.55±3.63 mm^3^ (SE), range = 20.2–44.3 mm^3^, N = 6), though these were somewhat smaller than the average CP volume of typical breeding males (mean = 119.29±2.5 mm^3^, N = 348). In contrast, control males did not develop a measurable CP during the course of the experiment. After implant removal all males developed CP's, and by the time of the post-treatment recapture CP volumes did not differ between T, control, and ASY males (Table 1, Fig. 1B). The first measureable CP found on an un-manipulated bird was observed on October 18, approximately 6 weeks after those seen in T males (on an ASY (age 4+ yrs) red/black male that had overwintered in the nuptial plumage). Thus, the induction of CP growth in T implanted males far preceded such sexual development in the population (see significant interaction between treatment and time on CP volume, Table 1).

Bill color differed with both treatment and time, darkening over the course of the experiment for all males (Table 1, Fig. 1C; see also Fig S1B). The darkening of T male bills followed a similar trajectory to that of un-manipulated ASY males (Fig. 1C) and at implant removal T and ASY male bill color did not differ (χ^2^ = 0.04, df = 1, p = 0.838), whereas control male bills were lighter than both T and ASY male bills at all capture periods (Fig. 1C). T male bill color did not increase after implants were removed (implant removal vs. post-treatment T male bill color; χ^2^ = 0.07, df = 1, p = 0.792) and at the post-treatment recapture T males trended towards having lighter bills than those of ASY males (χ^2^ = 3.16, df = 1, p = 0.076), albeit still darker than those of control males (χ^2^ = 4.67, df = 1, p = 0.031).

### Molt and Plumage Color

**Figure 2:**
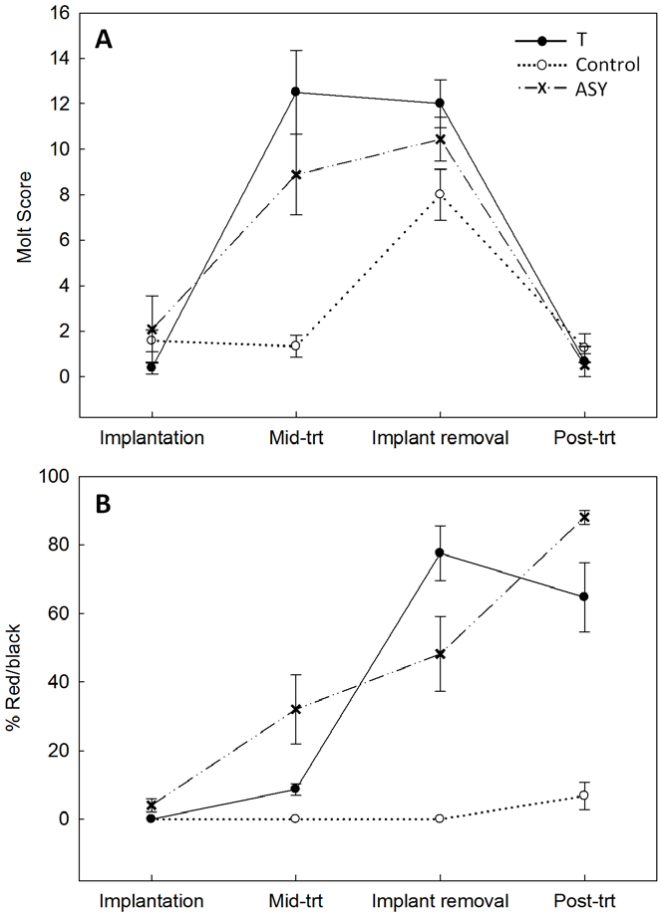
Treatment effects on molt score (A) and extent of nuptial plumage coloration (% red/black; B). Changes in mean response ± SE for second-year testosterone (T) and control implanted males, and unmanipulated after-second year (ASY) males at implantation, the mid-treatment recapture, implant removal, and the post-treatment recapture.

T treatment stimulated early onset of molt relative to control males (Table 1, Fig. 2A; see also Fig. S1C). Molt intensity differed with treatment at the mid-treatment recapture (χ^2^ = 9.26, df = 2, p = 0.01), but was similar across treatment types at implant removal (χ^2^ = 4.46, df = 2, p = 0.11), primarily because molt intensity was increasing in control and ASY males and T males were nearing completion of molt.

The extent of nuptial coloration (% red/black) obtained during molt differed significantly among treatments (Table 1, Fig. 2B; see also Fig. S1D): T males produced red/black plumage in all respective feather tracts, whereas control males produced the brown plumage coloration typical of the majority of SY males. Percent red/black differed between treatments at the post-treatment recapture (Table 1) with T males more similar to ASY than to control males (T vs. ASY: χ^2^ = 3.0, df = 1, p = 0.083; T vs. control: χ^2^ = 4.58, df = 1, p = 0.032). While T male % red/black did not change significantly between implant removal and post-treatment recapture (χ^2^ = 1.07, df = 1, p = 0.302; Fig. 2B), feathers plucked at the time of implant removal grew back brown in the absence of T-implants. In contrast, control males showed a trend for increase in % red/black (χ^2^ = 3.33, df = 1, p = 0.068; Fig. 2B), consequence of feather plucking as feathers grew back red/black on control birds that gained breeding positions (N = 3) and brown on the control bird that remained as a natal auxiliary (N = 1). All monitored T males acquired breeding positions (N = 3).

**Figure 3:**
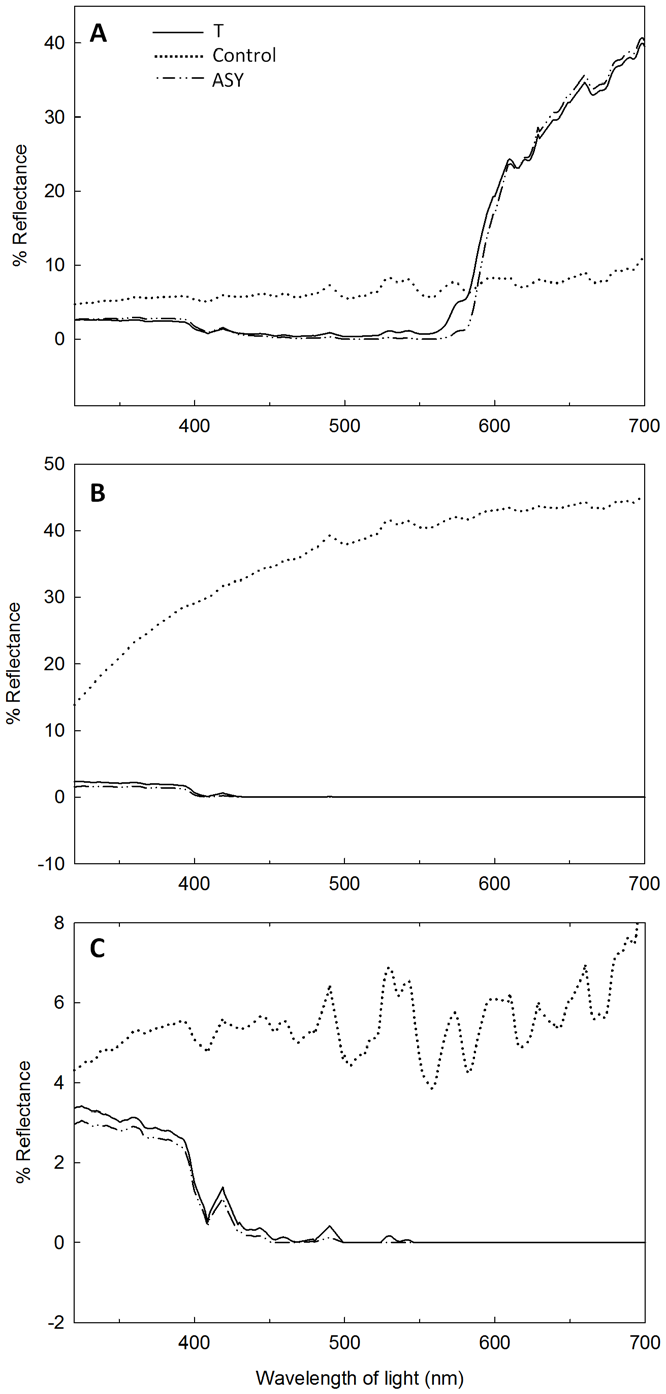
Reflectance spectra of back (A), breast (B), and crown (C) feathers. Lines indicate mean reflectance from testosterone (T, N = 6), control (N = 6), and after-second year (ASY, N = 4) males from whom feathers were collected at implant removal.

The spectral qualities of back, breast, and crown feathers varied with treatment, primarily as a consequence of the marked differences in reflectance parameters between the brown/white feathers of control and the red/black feathers of T and ASY males (Table 2, Fig. 3A–C; see also Fig. S3A–C). While T and ASY males did not differ in the reflectance parameters of back and crown feathers (Table 2, Fig. 3A,C), there were non-significant trends for differences in breast feather brightness (Table 2, Fig. 3B; T (mean±SE) 1.77±0.18; ASY 1.21±0.15) and hue (Table 2; T 330.29±4.21 nm, ASY 346.19±7.18 nm), and a significant difference in UV chroma with T males producing breast feathers having a lower UV chroma than those of ASY males (Table 2, Fig. S4; T 0.95±0.01, ASY 0.98±0.0.007).

**Table 2:** Summary of statistical differences in feather color at implant removal between treatment types (T, control) and unmanipulated (ASY) males in all measured color metrics and for three representative plumage patches (red, carotenoid-pigmented back feathers and black melanin-pigmented breast and crown feathers).

Color Metric (Model)	Back		Breast		Crown	
	χ^2^, df	P	χ^2^, df	p	χ^2^, df	p
Brightness (All^1^)	6.25, 2	**0.044**	12.1, 2	**0.002**	10.8, 2	**0.005**
Brightness(T vs. ASY^2^)	0.05, 1	0.831	3.68, 1	*0.055*	0.41, 1	0.522
Spectral Purity (All)	10.6, 2	**0.005**	11.8, 2	**0.003**	10.6, 2	**0.005**
Spectral Purity (T vs. ASY)	0.00, 1	1.000	2.91, 1	0.088	0.00, 1	1.000
Red Chroma (All)	10.8, 2	**0.005**	13.9, 2	**<0.001**	14.0, 2	**<0.001**
Red Chroma (T vs. ASY)	0.41, 1	0.522	0.00, 1	1.000	0.00, 1	1.000
UV Chroma (All)	11.3, 2	**0.004**	12.4, 2	**0.002**	10.8, 2	**0.005**
UV Chroma (T vs. ASY)	1.64, 1	0.201	4.54, 1	**0.033**	0.41, 1	0.522
Hue (All)	3.26, 2	0.195	12.2, 2	**0.002**	11.1, 2	**0.004**
Hue (T vs. ASY)	0.47, 1	0.494	3.68, 1	*0.055*	1.14, 1	0.286

Significant variables are depicted in bold and near significant (0.05<p<0.1) variables are italicized. For all relevant analyses N = 6 control, 6 T, and 4 ASY males.

1 Results from an analysis including “All” treatments; T, control, and ASY males.

2 Results from a pair-wise comparison of “T vs. ASY” males only.

## Discussion

We show that testosterone (T) is the primary mechanism functioning during the pre-nuptial molt to regulate variable plumage color and breeding phenotype in male red-backed fairy-wrens. Experimental elevation of T prior to breeding stimulated rapid and early onset molt and resulted in red/black nuptial plumage acquisition, bill darkening, and cloacal protuberance (CP) growth. Indeed, alterations induced by T in young (SY) male plumage color and morphology preceded but closely mirrored seasonal changes documented in un-manipulated older (ASY) males. T males produced back and crown feathers that did not quantitatively differ in reflectance parameters from those of ASY males, indicating that T is a sufficient stimulus for the production of “normal” red/black plumage (but see below). In contrast, control males acquired the brown plumage common to 85% of un-manipulated SY males, and temporal changes in molt intensity, bill color, and CP volume were slower than for T males, coinciding with reproductive readiness in the population. Removal of T implants led to a cessation in trait elaboration, and red/black feathers plucked from T males at implant removal were replaced with brown feathers, indicating that the effects of T are reversible and that T has to remain elevated during feather growth to induce red or black feather pigmentation. In contrast, feathers plucked at implant removal from control males grew back brown on one male who remained as natal auxiliary, but red/black on males who obtained a breeding position, a change that corresponded with a seasonal increase in fecal androgen metabolite concentrations. Our previous work has shown that post-molt changes in social status from auxiliary to breeder increased both androgen excretion and bill color in our study population, but had limited impact on plumage color [34]. Thus, correlational analyses [33], experimental manipulations of social status [34], and now experimental manipulations of T (this study) converge to provide strong and unequivocal evidence that male red-backed fairy-wren breeding morphology (CP volume, bill and plumage color) is activated by changes in testosterone, and that T levels in turn are associated with body condition [33]and social status [33], [34].

While T treatment induced SY males to produce feathers virtually indistinguishable from those of ASY males, slight differences in the reflectance parameters of breast feathers from T and ASY males, combined with the spectral qualities of feathers produced by Anti-T males (see Text S1, Table S1, and Fig. S3), suggest possible differences in the mechanisms controlling production of carotenoid- vs. melanin-pigment feathers. Despite variation in effective androgen levels and age, T, ASY, and Anti-T males produced spectrally similar red carotenoid-pigmented back feathers. In contrast, UV chroma and spectral purity of the presumably melanin-based black breast feathers differed with lower UV chroma, lighter hue and higher total reflectance in T vs. ASY males (Anti-T male breast and crown feathers differed from both T and ASY males and were more similar to control male coloration, see Fig. S3B,C). These differences could occur if the rapid molt induced in T males resulted in a less complete final coloration, or if factors related to age and independent of T affect production of the “normal” red/black plumage type, and in particular color of melanin-based breast plumage. Thus, while the carotenoid response to T is robust, melanin pigmentation (or alternatively nuptial pigmentation of the breast feather tract) appears to be sensitive to molt duration, to variable dosages of T, or to the cellular mechanisms of T action that are potentially related to age. Future studies are needed on this and other species to examine differences between age-classes, pigment types, and feather tracts in T dependence of plumage coloration. Specifically, more studies on within-individual effects of T on different patches and color elements will illuminate the generality and diversity of T effects on plumage color.

Red-backed fairy-wren plumage color is sexually selected, with red-black males having higher annual reproductive success than brown males [31], [32], thus raising the question of why males would breed in brown plumage [32]. Our results show that plumage color is related to variation in condition during molt [33], but despite the stimulatory effects of T on coloration (this study), the role of T in regulating signal honesty remains unclear. Recently several hypotheses have been proposed suggesting that the physiological costs of T are responsible for maintaining signal honesty [43], [61], [62]. For example, the “immunocompetance handicap hypothesis” [3] posits that, due to the immunosuppressive properties of T, only the most immunocompetent males can produce an elaborate signal without suffering T-related costs to health. For the red-backed fairy-wren, several results suggest that the physiological costs of T may play such a role: natural variation in condition of molting males is positively related to variation in T [33], experimental elevation of T is immunosuppressive in the congeneric Superb Fairy-wren [7], and T has costly impacts on metabolism [5] and behaviour [6] in other avian species. However, in this study we did not document any physiological costs to artificially elevated T (condition or corticosterone), T males acquired breeding positions, and experimental shifts in status from auxiliary to breeder result in immediate increases in excreted androgens [34]. Together, these results suggest that social status and not T constrains signal development; relief of social pressure enables increases in T and induction of T-dependent signals without impacting condition or impairing ability to successfully obtain mates and breed (this study, [34]). Studies of the relationship between immune function, other physiological parameters (e.g. metabolism, oxidative stress), and naturally circulating as well as artificially elevated T are now needed to determine whether T carries any costs that would contribute to the maintenance of signal honesty in red-backed fairy-wrens.

The results presented here, in combination with those of [22] on a congeneric Malurid, demonstrate direct effects of T on plumage color during a pre-nuptial molt occurring prior to breeding when gonads are active. Although it is sometimes true that sexually selected variation in passerine plumage color reflects T during breeding [16], [63]–[65], acquisition of male-typical nuptial plumage is generally thought to be independent of T in this large taxonomic group [14]. However, the majority of species for which this mechanism has been investigated acquire nuptial plumage during a post-nuptial molt occurring at a time when T secretion is low [18], [66]. In many of these species, T delays or prevents molt [18]–[20], [25]–[28], leads to the production of drab plumage [20], or has no observable effect on plumage color [21]. In other species where males acquire nuptial plumage during a post-nuptial molt, intrasexual variation in plumage color is regulated by indirect effects of T acting outside of the molting period, for example through organizational events acting early in development [29], [30], [67] and effects on behaviour (e.g., increased preening; [16], [17], [68]). Thus, T can have both direct and indirect effects on plumage coloration in passerines, with evidence to date suggesting that direct effects occur in species with a pre-nuptial molt that occurs when T is elevated ([22], [33], this study). Future studies are needed on other passerine species with a pre-nuptial molt to test this hypothesis.

## Supporting Information

Figure S1 **Treatment effects on fecal androgens (A), bill color (B), molt (C), and % red/black (D).** Changes in mean response ± SE for second-year testosterone (T), control, and anti-testosterone (Anti-T) implanted males at implantation, the mid-treatment recapture, implant removal, and the post-treatment recapture.
(TIF)

Figure S2 **Changes in body condition of testosterone, control, and after-second year males.** Changes in mean residual body condition ± SE at implantation, the mid-treatment recapture, implant removal, and the post-treatment recapture.
(TIF)

Figure S3 **Reflectance of testosterone, control, and anti-testosterone back (A), breast (B), and crown (C) feathers.** Lines indicate mean reflectance from testosterone (T, N = 6), control (N = 6), and Anti-T males (N = 5) from whom feathers were collected at implant removal.
(TIF)

Figure S4 **UV chroma of testosterone and after-second year male breast feathers.** Mean UV chroma ± SE of breast feathers collected at implant removal from T (N = 6) and ASY males (N = 4).
(TIF)

Figure S5 **Plasma luteinizing hormone (LH) concentrations of testosterone, anti-testosterone, control, and after-second year males.** Bars indicate treatment mean LH concentrations ± SE from plasma collected at the mid-treatment recapture.
(TIF)

Text S1 **Anti-testosterone treatment; methods, results, and a discussion of the mechanisms of nuptial plumage production.**
(DOC)

Table S1 **Variation between testosterone, anti-testosterone and after-second-year males in reflectance of back, breast and crown feathers.**
(DOC)
