# Supplementary figures and images for: Sexually Selected Male Plumage Color Is Testosterone Dependent in a Tropical Passerine Bird, the Red-Backed Fairy-Wren (Malurus melanocephalus)
Source: PLoS One. 2011 Oct 5;6(10):e26067. doi: 10.1371/journal.pone.0026067 (PMC3187837; doi:10.1371/journal.pone.0026067)

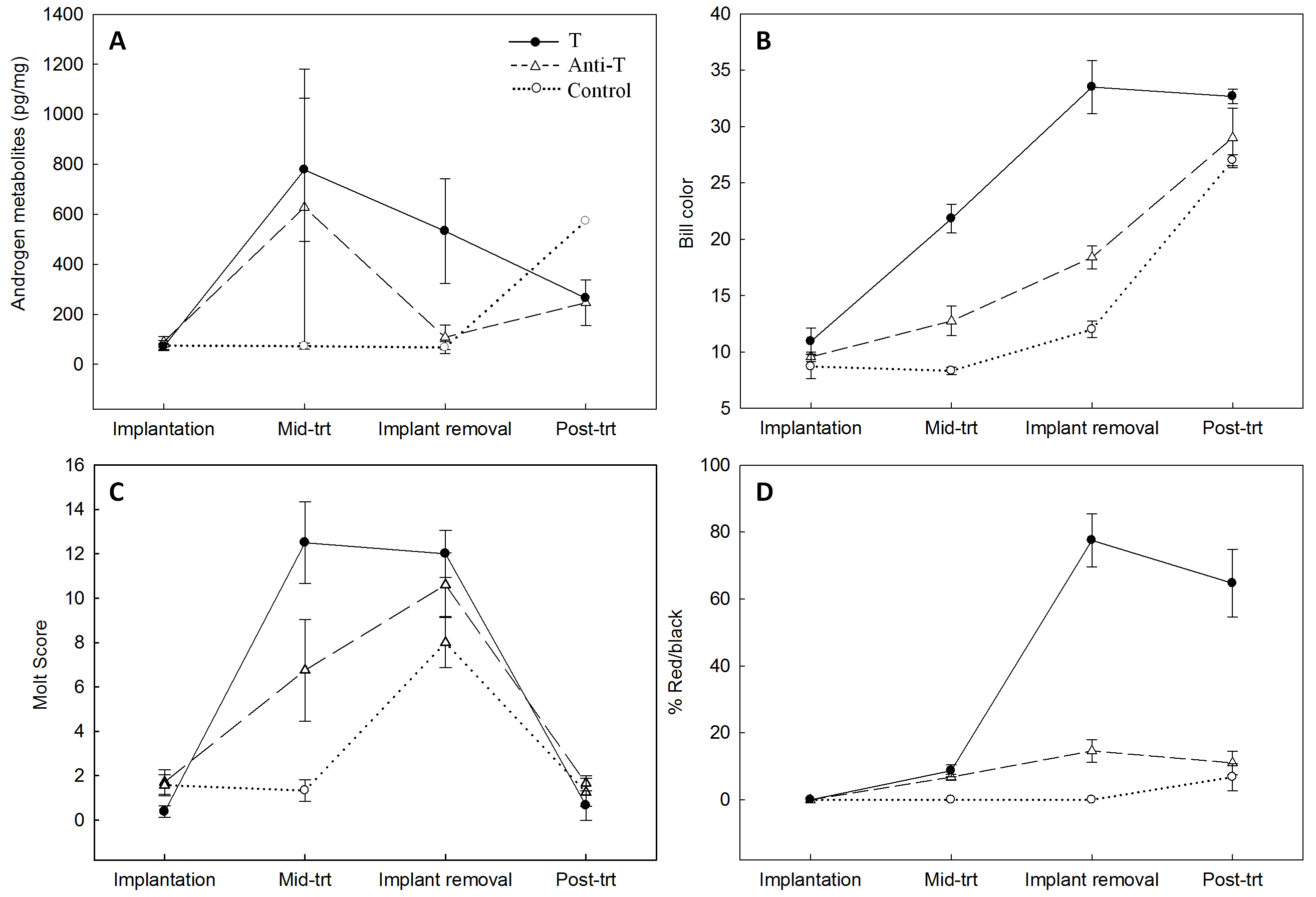

Supplement: Figure S1 — Treatment effects on fecal androgens (A), bill color (B), molt (C), and % red/black (D). Changes in mean response ± SE for second-year testosterone (T), control, and anti-testosterone (Anti-T) implanted males at implantation, the mid-treatment recapture, implant removal, and the post-treatment recapture. (TIF) [file pone.0026067.s001.tif]

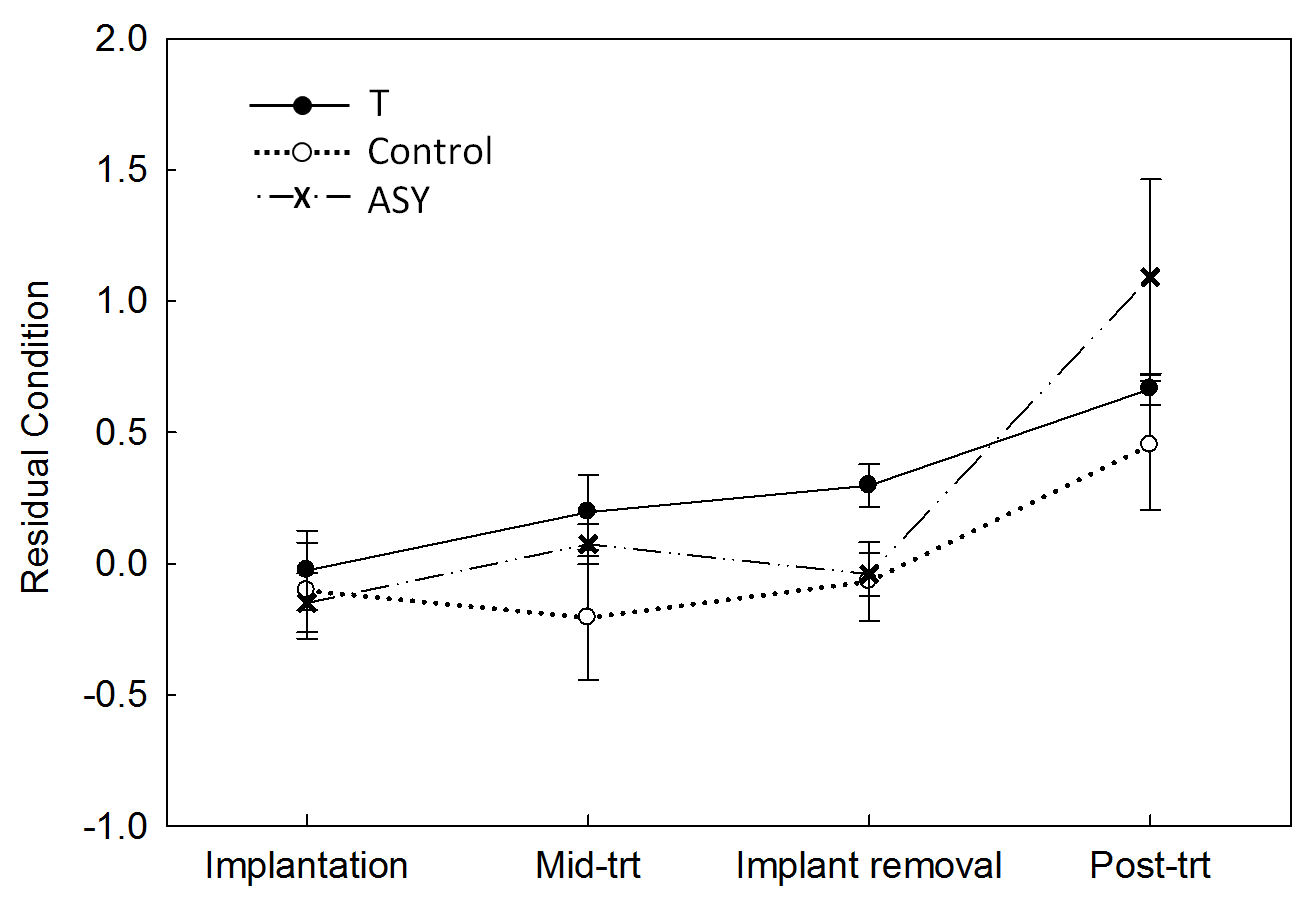

Supplement: Figure S2 — Changes in body condition of testosterone, control, and after-second year males. Changes in mean residual body condition ± SE at implantation, the mid-treatment recapture, implant removal, and the post-treatment recapture. (TIF) [file pone.0026067.s002.tif]

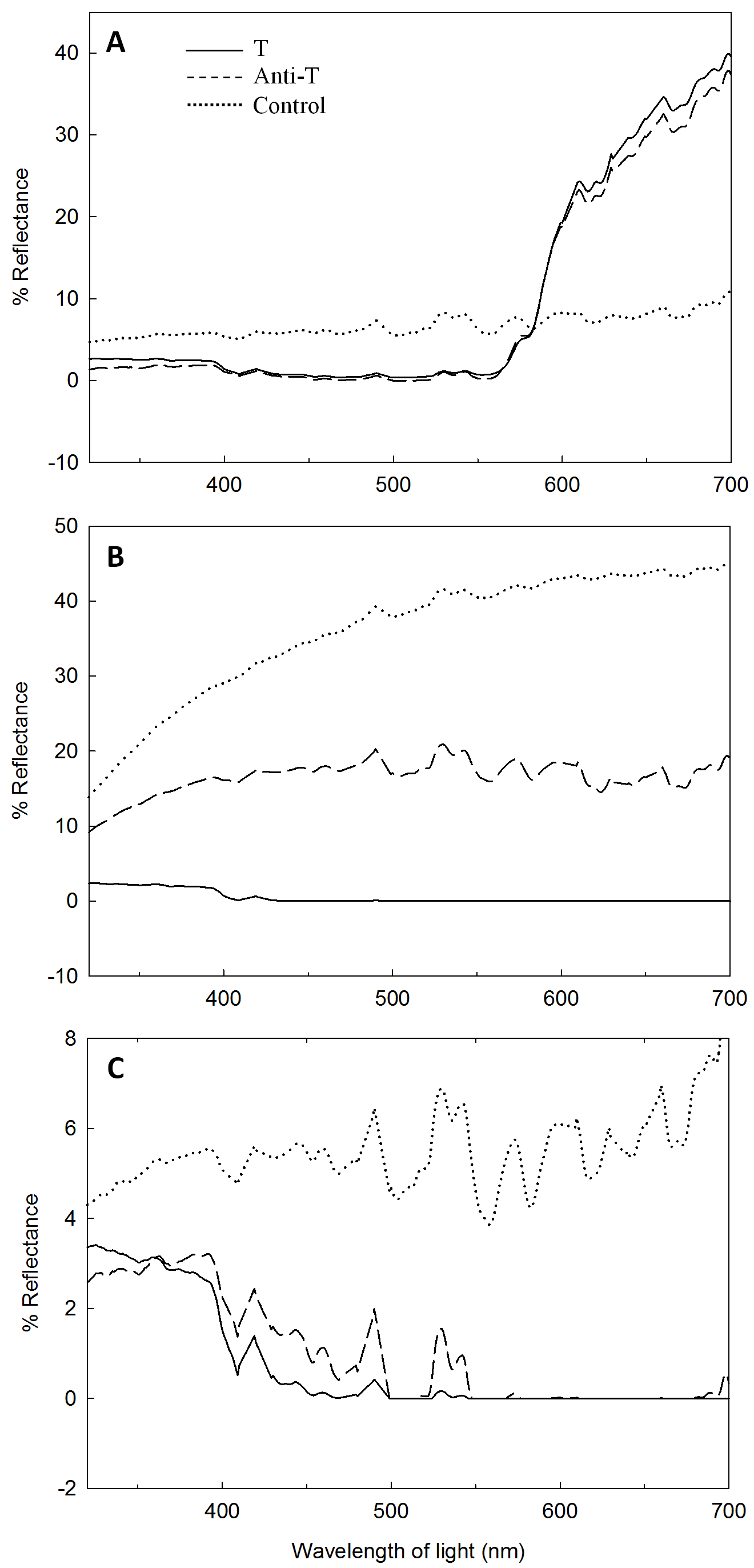

Supplement: Figure S3 — Reflectance of testosterone, control, and anti-testosterone back (A), breast (B), and crown (C) feathers. Lines indicate mean reflectance from testosterone (T, N = 6), control (N = 6), and Anti-T males (N = 5) from whom feathers were collected at implant removal. (TIF) [file pone.0026067.s003.tif]

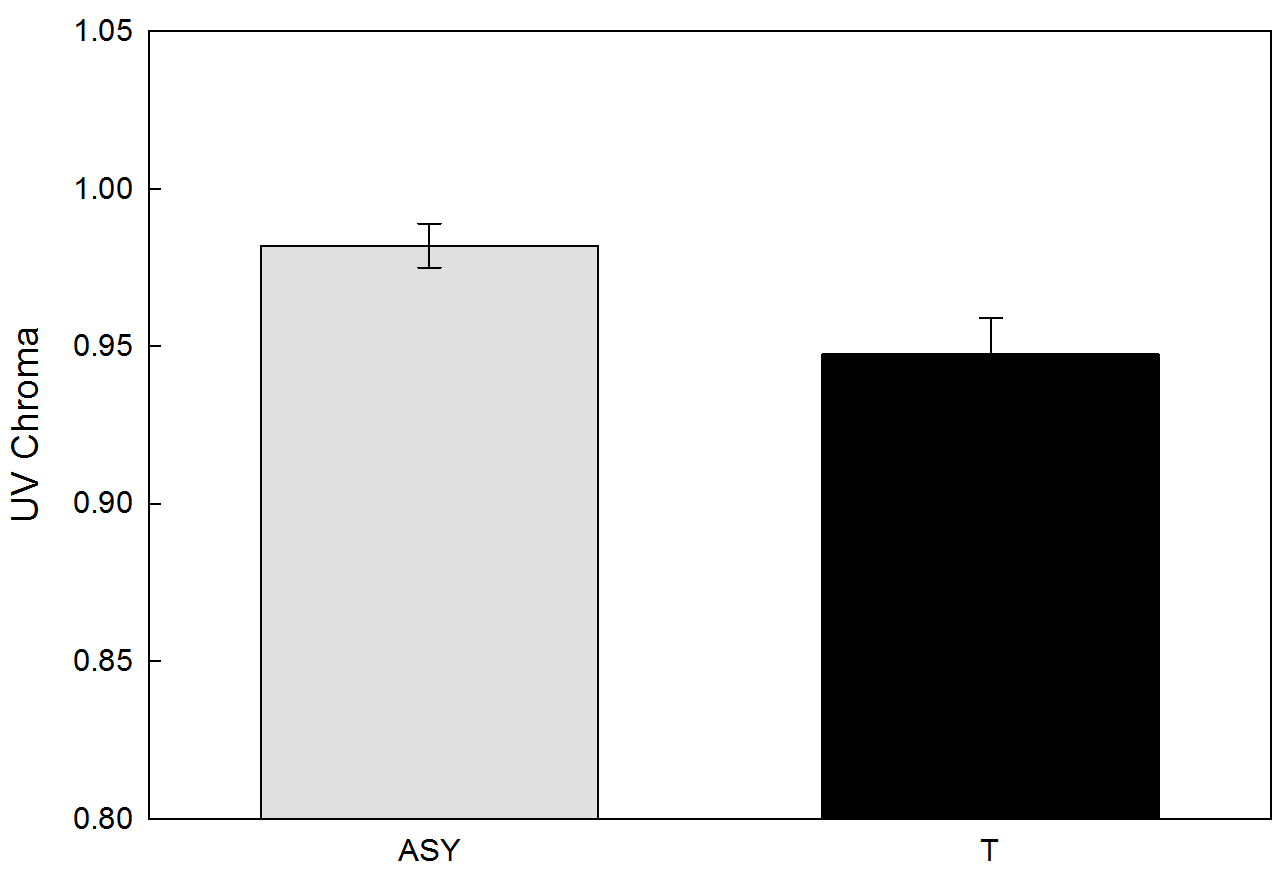

Supplement: Figure S4 — UV chroma of testosterone and after-second year male breast feathers. Mean UV chroma ± SE of breast feathers collected at implant removal from T (N = 6) and ASY males (N = 4). (TIF) [file pone.0026067.s004.tif]

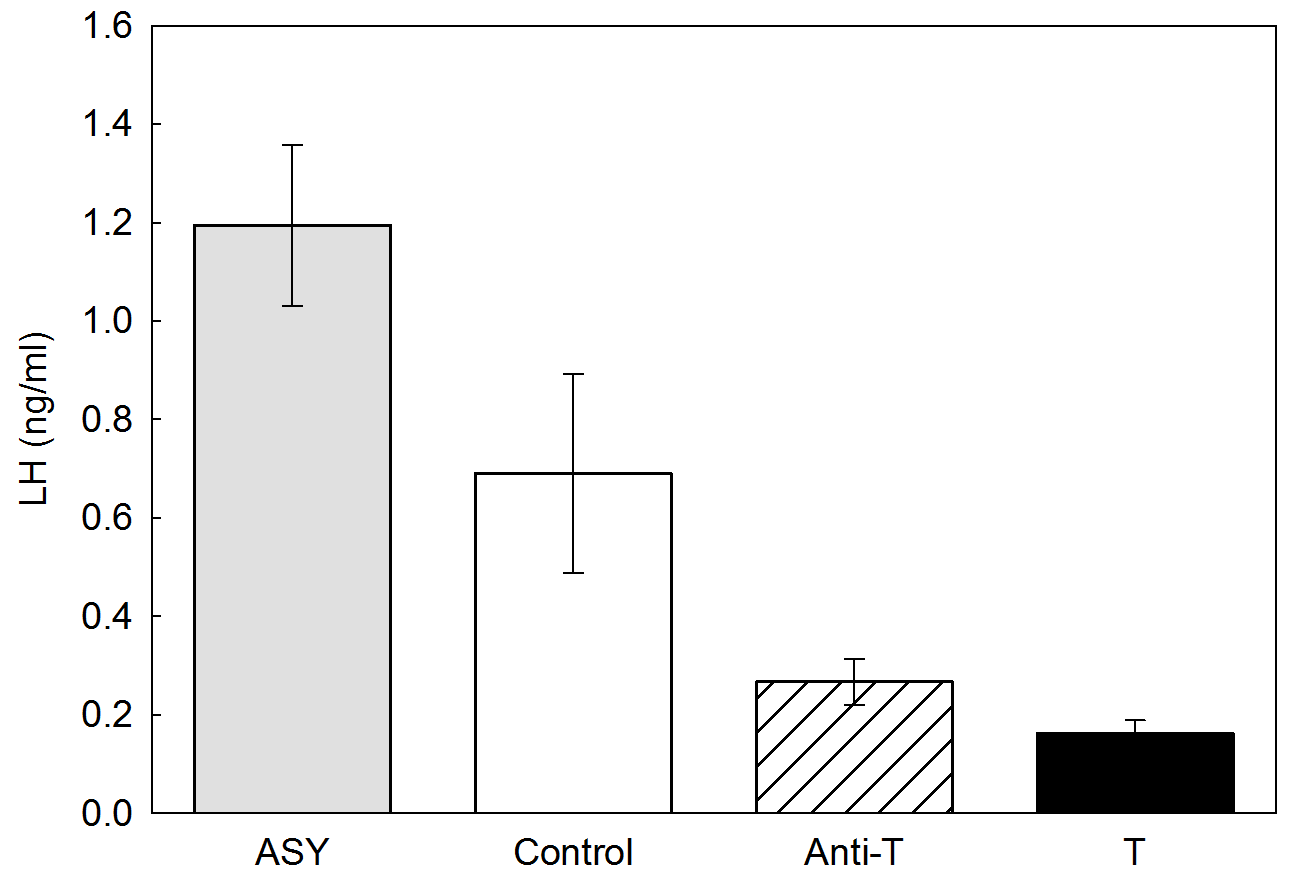

Supplement: Figure S5 — Plasma luteinizing hormone (LH) concentrations of testosterone, anti-testosterone, control, and after-second year males. Bars indicate treatment mean LH concentrations ± SE from plasma collected at the mid-treatment recapture. (TIF) [file pone.0026067.s005.tif]
