# Supplementary material for: Sexually Selected Male Plumage Color Is Testosterone Dependent in a Tropical Passerine Bird, the Red-Backed Fairy-Wren (Malurus melanocephalus)
Source: PLoS One. 2011 Oct 5;6(10):e26067. doi: 10.1371/journal.pone.0026067 (PMC3187837; doi:10.1371/journal.pone.0026067)
Supplement: Text S1 — Anti-testosterone treatment; methods, results, and a discussion of the mechanisms of nuptial plumage production. (DOC) [file pone.0026067.s006.doc]

**Anti-testosterone treatment; methods, results, and a discussion of the mechanisms of nuptial plumage production**

**Methods**

The effects of T on behaviour and morphology can result from the actions of androgens via the androgen receptor pathway (including effects after conversion of T to 5α-dihydrotestosterone by 5α-reductase), or can stem from enzymatic conversion of T to estradiol by aromatase and subsequent actions via the oestrogen receptor pathway. Therefore, our anti-testosterone (Anti-T) treatment consisted of a combination of an aromatase inhibitor (blocking T conversion into 17β-estradiol) and an anti-androgen (androgen receptor antagonist) to block both known pathways by which T might affect plumage color. Specifically, we used 1-4-6 androstatrien-3,17dione (ATD; Sigma A7710), which is an effective, irreversible aromatase inhibitor [1], and trifluoro-2-methyl-4’-nitro-m-propionotoluidid (flutamide; Sigma F9397), which is a reversible androgen antagonist that blocks androgen receptors. Previous studies of songbirds have combined ATD and flutamide to effectively block actions of testosterone [2-6].

Luteinizing Hormone (LH) Radioimmunoassay

In consequence of the surprising effects of our Anti-T treatment on plumage color and morphology (see below), we conducted a post-hoc analysis of plasma LH concentration utilizing plasma collected at the mid-treatment recapture. We analyzed LH concentration in plasma of Anti-T (N=3), T (N=6), control (N=6), and unmanipulated ASY males molting into red/black plumage (N=5). We measured LH by a post-precipitation, double-antibody radioimmunoassay [7, 8]. This assay utilizes purified chicken LH for standard curves and for radio-iodination and goat anti-rabbit gamma globulin precipitating serum as second antibody. Further details of the LH assay are described by [9], and intra- and inter-assay variabilities were similar to those of previous studies.

**Results**

While we had near perfect implant retention for T and control implants for the duration of the experiment (1 control implant was lost), the Anti-T implants were all lost within 2 weeks of implantation (by the mid-treatment recapture). As a result, only one of the 4 Anti-T males sampled at the mid-treatment recapture had retained both implants, one had lost both implants, and the other two had only the flutamide implant remaining. By implant removal, none of the birds had retained Anti-T implants (N=4 recaptured birds).

Androgens and Luteinizing Hormone

Anti-T males had intermediate concentrations of excreted androgens at the mid-treatment recapture that did not differ from either T (Fig. S1A; χ^2^=1.07, df=1, p=0.302) or control males (χ^2^=0.5, df=1, p=0.479), but at implant removal their androgen metabolite concentrations were basal, albeit not significantly lower than those of T males (Fig. S1A; χ^2^=1.35, df=1, p=0.245). The increase and decline of excreted androgen metabolite concentrations of Anti-T males between implantation and implant removal corresponded with implant loss.

LH concentration at the mid-treatment recapture differed with treatment (Fig. S5; ANOVA conducted using log-linearized LH concentrations: F_2,12_=13.44, p=0.0009). As predicted by negative feedback inhibition of T on LH secretion, T implanted males had significantly lower concentrations of LH than control, Anti-T, and unmanipulated ASY males (Tukey adjustment for multiple comparisons, α=0.05; control, Anti-T, and ASY male LH did not differ). There was no apparent difference in LH concentration between Anti-T males which had retained both the ATD and the flutamide implant and those with only the flutamide implant remaining (N=3; low sample sizes precluded statistical analysis).

CP Volume and Bill Color

No Anti-T males developed a visible CP during the course of the experiment, but by the time of the post-treatment recapture CP volumes did not differ between the treatment groups (T, Anti-T, and control) and unmanipulated red/black males (χ^2^=0.436, df=3, p=0.9327).

At the mid-treatment recapture the bills of T males were not significantly darker than those of Anti-T males (Fig. S1B; χ^2^=2.72, df=1, p=0.099), but Anti-T males had significantly darker bills than did control males (Anti-T vs. control: χ^2^=7.71, df=1, p=0.006). All males exhibited bill darkening over the course of the treatment period and by the post-treatment recapture there was no difference in bill color between T, Anti-T, and control males (χ^2^=3.97, df=2, p=0.138)

Molt and Plumage Color

Anti-T treatment stimulated early onset of molt (Fig. S1C) compared to controls, and Anti-T molt intensity at the mid-treatment recapture was intermediate; molt intensity was not significantly lower than in T males but higher than in control males (Anti-T vs. T: χ^2^=2.59, df=1, p=0.108; Anti-T vs. control: χ^2^=4.71, df=1, p=0.029).

Anti-T males exhibited an initial increase in % red/black plumage that paralleled that seen in T males and was different from that of control males (Fig. S1D; mid-treatment Anti-T vs. T: χ^2^=1.39, df=1, p=0.238; Anti-T vs. control: χ^2^=8.31, df=1, p=0.004); but the acquisition of red/black coloration in Anti-T males was truncated at the mid-treatment recapture and % red/black of Anti-T males was significantly lower than for T males at implant removal, but still greater than for control males (Anti-T vs. T: χ^2^=7.5, df=1, p=0.006; Anti-T vs. control: χ^2^=8.91, df=1, p=0.003). As with T males, red/black feathers plucked from Anti-T males at implant removal grew back brown.

While Anti-T males produced red back feathers that were spectrographically similar to those of T and ASY males (Table S1, Fig S3A), the coloration of their breast feathers differed dramatically (Table S1, Fig S3B); Anti-T birds produced dark brown, not black breast feathers with a significantly lower spectral purity than in T and ASY males (α=0.05; Anti-T (mean ± SE) 1.32±0.48, T 5.39±0.10, ASY 5.72±0.17), higher red chroma (Anti-T 0.19±0.05, T 0±0, ASY 0±0), lower UV chroma (Anti-T 0.26±0.08, T 0.95±0.01, ASY 0.98±0.0.007), and highest hue (Anti-T 526.55±46.59nm, T; 330.29±4.21 nm, ASY 346.19±7.18 nm). Anti-T, T, and ASY males all produced similar colored black crown feathers with the exception that crown color produced by Anti-T birds was of a significantly higher hue (Table S1, Fig S3C; Anti-T 399±24.89 nm, T 325.04±1.17 nm, ASY 328.56±3.09 nm); there was a trend for lower spectral purity in the crown feathers of Anti-T males vs. those of T and ASY males (Anti-T vs ASY: χ^2^=3.84, df=1, p=0.05; Anti-T vs T: χ^2^=4.03, df=1, p=0.045).

**Discussion**

The results of our Anti-T treatment, designed to rigorously test for a role of T in regulation of plumage phenotype by pharmacological blockage of T action, were unexpected and surprising but also informative. Our pharmacological approach, while failing due to implant loss, reveals our current ignorance about the physiological mechanisms of sexual signal production. Anti-T treated males acquired plumage intermediate in color to that of T and control males, and also demonstrated bill darkening and an early onset of molt. Three scenarios might explain these results, all of which pertain to cellular mechanisms of plumage color control. 1) The inability of Anti-T birds to perceive T due to blocking of both the androgen- and the oestrogen-receptor pathway should have disrupted negative feedback on the hypothalamus and pituitary and lead to heightened LH secretion. We hypothesized that increased LH stimulated feather follicles to produce elaborate nuptial plumage (as seen in other passerine species, but see “results” and below). 2) The flutamide treatment may have partially failed or malfunctioned (incomplete androgen receptor antagonism or receptor agonism). 3) The ATD implant may have been ineffective allowing for conversion of T to oestradiol and explaining effects on trait elaboration only if those traits are controlled by T functioning on oestrogen receptors after conversion by aromatase.

Molt into sexually dichromatic nuptial plumage is stimulated by LH in most passerine species studied to date ([10]; reviewed in [11]; but see [12])]. In these species heightened concentrations of oestradiol in females are assumed to suppress LH secretion and hence impede development of a male-like nuptial coloration, while males produce elaborate plumage in the presence of androgens as well as following gonadectomy [10]. We conducted post-hoc plasma LH assays from samples collected at the mid-treatment recapture, predicting that concentrations of LH in Anti-T birds would be elevated compared to control and T treated males. Contrary to our predictions, there was no difference in plasma LH between Anti-T, control, and ASY males. However, T males had the lowest concentrations of LH as would arise from strong negative feedback on the brain and pituitary. Thus, we find no evidence to support a role of LH in acquisition of male nuptial plumage coloration in red-backed fairy wrens. Moreover, the intermediate concentrations of LH expressed by Anti-T males are suggestive of flutamide malfunction, enabling partially effective negative T feedback on the pituitary and limiting LH secretion.

Additional evidence supports the possibility of malfunctioning androgen receptor antagonism by flutamide: Anti-T male fecal androgen metabolite concentrations were elevated at the mid-treatment recapture (prior to implant loss), their bills darkened earlier, and molt intensity was heightened relative to controls. This suggests that T was functional (excreted T metabolites were elevated) and the flutamide ineffective. While flutamide has performed reliably as an androgen receptor antagonist in other experimental studies of birds [2-6], it can also upregulate androgen receptor expression (mRNA) and can cause time-dependent insensitivity to further antagonism [13, 14]. However, Anti-T males did not produce a measurable CP during the period of their elevated androgens, suggesting either that CP growth requires a higher initial threshold of T than bill color and onset of molt, or indicating that flutamide was indeed effective and the ATD implant failed. In this third scenario, local conversion of T to oestradiol would explain the bill darkening and enhanced molt of the Anti-T males and also explain why CP’s did not develop in Anti-T males (assuming CP growth is androgen-dependent).

In sum, our attempt to block effects of endogenous T at the level of the target cell was clearly ineffective, both due to implant loss as well as malfunction or unexpected pharmacological effects of either the ATD or flutamide treatments. These results highlight the limitations to our understanding of the cellular mechanisms of T action on feather follicles and other traits. Specifically, T may interact with androgen receptors in feather follicles to stimulate pigment production, transport and deposition or may function via the oestrogen receptor after aromatase conversion. Rigorous and carefully controlled pharmacological approaches are now necessary before we can conclude that T, and only T, regulates male sexual signal production at the cellular level.

**References**

1. Foidart A, Tlemcani O, Harada N, Abe-Dohmae S, Balthazart J (1995) Pre- and post-translational regulation of aromatase by steroidal and non-steroidal aromatase inhibitors. Brain Res 701: 267-278.
2. Schwabl H, Kriner E (1991) Territorial aggression and song of male European robins (*Erithacus rubecula*) in autumn and spring: effects of antiandrogen treatment. Horm Behav 25; 180-194.
3. Soma KK, Sullivan K, Wingfield JC (1999) Combined aromatase inhibitor and antiandrogen treatment decreases territorial aggression in a wild songbird during the nonbreeding season. Gen Comp Endocrinol 115; 442-453.
4. Canoine V, Gwinner E (2002) Seasonal differences in the hormonal control of territorial aggression in free-living european stonechats. Horm Behav 41; 1-8.
5. Moore IT, Walker BG, Wingfield JC (2004) The effects of combined aromatase inhibitor and anti-androgen on male territorial aggression in a tropical population of rufous-collared sparrows, *Zonotrichia capensis*. Gen Comp Endocrinol 135; 223-229.
6. Van Duyse E, Pinxten R. Snoeijs T, Eens M (2005) Simultaneous treatment with an aromatase inhibitor and an anti-androgen decreases the likelihood of dawn song in free-living male great tits, *Parus major*. Horm Behav 48; 243-251.
7. Follett BK, Scanes CG, Cunningham FJ (1972) A radioimmunoassay for avian luteinizing hormone. J Endocrinol 52; 359-378.
8. Sharp PJ, Dunn IC, Talbot RT (1987) Sex differences in response to chicken LHRH-I and II in the domestic fowl. J Endocrinol 115; 323-331.
9. Wingfield JC, Hegner RE, Lewis D (1991) Circulating levels of luteinizing hormone and steroid hormones in relation to social status in the cooperatively breeding white-browed sparrow weaver, *Plocepasser mahali*. J Zool Lond 225; 43-58.
10. Witschi E (1961) Sex and secondary sexual characters. In: Marshal AJ editor. Biology and comparative physiology of birds. New York: Academic Press. pp. 115-168.
11. Kimball RT (2006) Hormonal control of coloration. In: Hill GE, McGraw KJ editors. Bird Coloration Vol. 1. Mechanisms and Measurements. Cambridge, Massachusetts: Harvard University Press. pp. 431-468.
12. Peters A, Astheimer LB, Boland CRJ, Cockburn A (2000) Testosterone is involved in acquisition and maintenance of sexually selected male plumage in superb fairy-wrens, *Malurus cyaneus*. Behav Ecol Sociobiol 47: 438-445.
13. Chen CD, Welsbie DS, Tran C, Baek SH, Chen R, et al. (2004) Molecular determinants of resistance to antiandrogen therapy. Nature Med 10; 33-39.
14. Fusani L, Day LB, Canoine V, Reinemann D, Hernandez E, Schlinger BA (2007) Androgens and the elaborate courtship behaviour of a tropical lekking bird. Horm Behav 51; 62-68.
