# Supplementary material for: Sexually Selected Male Plumage Color Is Testosterone Dependent in a Tropical Passerine Bird, the Red-Backed Fairy-Wren (Malurus melanocephalus)
Source: PLoS One. 2011 Oct 5;6(10):e26067. doi: 10.1371/journal.pone.0026067 (PMC3187837; doi:10.1371/journal.pone.0026067)
Supplement: Table S1 — Variation between testosterone, anti-testosterone and after-second-year males in reflectance of back, breast and crown feathers. (DOC) [file pone.0026067.s007.doc]

**Table S1.** Variation between treatments (Anti-T, T, ASY) in reflectance parameters of back (red, carotenoid-pigmented), breast and crown feathers (black, melanin-pigmented).

| **Color metric** | **Back** | | **Breast** | | **Crown** | |
| --- | --- | --- | --- | --- | --- | --- |
|  | χ^2^, df | p | χ^2^, df | p | χ^2^, df | p |
| Brightness | 1.86, 2 | 0.395 | 11.1, 2 | **0.004** | 1.46, 2 | 0.483 |
| Spectral purity | 0.36, 2 | 0.829 | 10.7, 2 | **0.005** | 5.42, 2 | 0.067 |
| Red Chroma | 0.62, 2 | 0.733 | 9.88, 2 | **0.007** | 2.00, 2 | 0.368 |
| UV Chroma | 4.08, 2 | 0.130 | 11.5, 2 | **0.003** | 4.11, 2 | 0.128 |
| Hue | 0.82, 2 | 0.662 | 11.1, 2 | **0.004** | 9.89, 2 | **0.007** |

Reflectance parameters that vary significantly between treatments are indicated in bold.

N=6 control, 6 T, 5 Anti-T, and 4 ASY males.
